# Effects of pruning on mineral nutrients and untargeted metabolites in fresh leaves of *Camellia sinensis* cv. Shuixian

**DOI:** 10.3389/fpls.2022.1016511

**Published:** 2022-10-13

**Authors:** Yang Liu, Jing Tian, Bei Liu, Zuopin Zhuo, Chen Shi, Ruineng Xu, Maoxing Xu, Baoshun Liu, Jianghua Ye, Lili Sun, Hong Liao

**Affiliations:** ^1^ Root Biology Center, College of Resources and Environment, Fujian Agriculture and Forestry University, Fuzhou, China; ^2^ Wuyi Mountain Tea Industry Research Institute, Wuyishan, China

**Keywords:** pruning, fresh tea leaf, mineral nutrient, metabolite, calcium, magnesium

## Abstract

Pruning is an important strategy for increasing tea production. However, the effects of pruning on tea quality are not well understood. In this study, tea leaves were collected from Wuyi Mountain for both ionomic and metabolomic analyses. A total of 1962 and 1188 fresh tea leaves were respectively collected from pruned and unpruned tea plants sampled across 350 tea plantations. Ionomic profiles of fresh tea leaves varied significantly between pruned and unpruned sources. For tea plants, pruning was tied to decreases in the concentrations of mobile elements, such as nitrogen (N), phosphorus (P), potassium (K) and magnesium (Mg), and dramatic increases in the concentrations of the immobile ions calcium (Ca), aluminum (Al), manganese (Mn), boron (B) and cobalt (Co). Clustering and heatmap analysis showed that pruning also affected tea leaf metabolism. Among 85 metabolites that were significantly impacted by pruning, 30 were identified through random forest analysis as characteristic differential metabolites with a prediction rate of 86.21%. Redundancy analysis showed that pruning effects on mineral nutrient concentrations accounted for 25.54% of the variation in characteristic metabolites between treatments, with the highest contributions of 6.64% and 3.69% coming from Ca and Mg, respectively. In correlation network analysis, Ca and Mg both exhibited close, though opposing correlations with six key metabolites, including key quality indicators 1,3-dicaffeoylquinic acid and 2-O-caffeoyl arbutin. In summary, large scale sampling over hundreds of tea plantations demonstrated that pruning affects tea quality, mainly through influences on leaf mineral composition, with Ca and Mg playing large roles. These results may provide a solid scientific basis for improved management of high-quality tea plantations.

## Introduction

Tea plants (*Camellia sinensis*) are the source of one of the most popular non-alcoholic beverages in the world (Xia et al., 2017). The pleasant taste and aroma of tea are mainly contributed by leaf metabolites, including polyphenols, amino acids, caffeine, aromatic compounds and organic acids (Ho et al., 2015; Das et al., 2019). Mineral nutrients not only play vital roles in the synthesis of tea metabolites, but also are themselves essential nutrients for human health (Malik et al., 2008; Salahinejad and Aflaki, 2010). Pruning is an important cultivation and management strategy that is widely employed to increase tea yields (Chen et al., 2021). Despite wide implementation, the effects of pruning on tea quality are still largely unknown. Thus, exploring the effects of pruning on the mineral and quality components of tea is informative for numerous tea producers, and may prove useful for tailoring management practices to improve tea quality.

Tea quality is affected by many factors, such as variety, environmental conditions, cultivation practices, and processing methods (Chen et al., 2018; Dong et al., 2019; Rubel Mozumder et al., 2021; Liu et al., 2022). Pruning tea plants several times per year is a common and useful management strategy that is widely applied across tea plantations in order to increase tea plant branching and productivity (Mohale et al., 2018). Previous studies have shown that pruning can promote the synthesis of indole-3-acetonitrile and menaquinone in branches, as well as, regulate indole-3-acetic acid and tryptophan metabolism, which, taken together, act to comprehensively promote branch development and growth (Arkorful et al., 2020b). Sun et al. (2018) explored the impacts of pruning on catechins in four varieties of tea plants, and found that the content of epigallocatechin gallate (EGCG) increased significantly with pruning, which would enhance tea bitterness and astringency. Some arbor-type tea plants are grown without pruning in order to maintain the original tea taste. Chen et al. (2021) explored Dancong tea quality and found that total amino acid content and chlorophyll content of tea leaves increased significantly when pruning was withheld, while the content of catechins decreased significantly. In the same study, the concentrations of aromatic compounds with floral and honey aromas were also significantly higher in tea not subjected to pruning, with notable examples including methyl salicylate, benzyl alcohol, benzeneacetaldehyde, jasmine lactone and trans-nerolidol (Chen et al., 2021).

At present, the effects of pruning on tea mineral nutrient and secondary metabolite contents remain largely unclear. In this study, global analysis of pruning effects on tea leaves was conducted through applying ionomic and non-targeted metabolomic technologies to explore impacts on mineral nutrient and secondary metabolite concentrations. Any conclusive results generated might provide a scientific basis for improving management of high-quality tea plantations through elucidation of pruning effects on tea leaf regulatory mineral elements and characteristic differential metabolites.

## Materials and methods

### Collection and processing of samples

In this study, an arbor-type tea variety (*Camellia sinensis* L. cv. “Shuixian”) was selected for treatment and observation. A total of 350 tea plantations in the main planting areas of Wuyi rock tea around Wuyishan City, Fujian, China were randomly selected in April, 2019 for sample collection. The geographic location information of the tea plantation is shown in **Supplementary Figure 1**. Tea plantations were divided into two types, which were 218 with pruned plants and 132 with unpruned plants.

Six representative tea plantations that included both pruned and unpruned tea plants were located in Huangbai Village, Wuyishan Scenic Area, Daoshuikeng, Matouyan, Wusandi and Chengdun Village (Sites 1-6). All sampled tea plants were between 20~30 years old.

Fresh tea leaves were collected by plucking according to local standards in ratios of three old leaves for each new leaf which is also called resting bud. A 10 g fresh leaf sample was collected from each tea plant. Nine tea plants were randomly selected from each tea plantation as biological replicates. A total of 1962 pruned tea leaves and 1188 unpruned tea leaves were picked across plantations. Seven replicates were heat-fixed at 105°C for 30 min, dried at 75°C to constant weights, and pulverized in a sample mill prior to conducting ionomic analysis. Two replicates were dried, pulverized and stored in a -20°C freezer for subsequent determination of untargeted metabolites.

Soil samples were obtained by first removing litter and leaves from the soil surface, and then collecting soil from 0-20 cm deep at the outer edge of the tree crown. Soil from the base of three plucked tea plants was mixed to form a single soil sample, and three replicated soil samples were taken from each tea plantation.

Soil samples were air-dried and ground through 2 mm and 0.149 mm sieves for determination of soil pH, organic matter (OM), available nitrogen (AN), available phosphorus (AP) available potassium (AK), exchangeable calcium (E-Ca) and exchangeable magnesium (E-Mg) through methods described by Bao (2000). Briefly, to determine organic matter and available nitrogen, high temperature potassium dichromate oxidation and alkaline hydrolysis diffusion methods were used, respectively. Available phosphorus was extracted by the Bray I method, while available potassium, exchangeable calcium and exchangeable magnesium were extracted in ammonium acetate solution and measured in a inductively coupled plasma-optical emission spectrometer (ICP-OES Avio 200, PerkinElmer, Waltham, US).

### Tea leaf ionomics

Mineral elements were detected in tea leaf samples after first digesting in H_2_SO_4_-H_2_O_2_ as previously described (Peng et al., 2018). The concentrations of nitrogen (N), phosphorus (P), and potassium (K) in the digestion solution were detected in a flow autoanalyzer (SKALAR SAN++, Skalar, Breda, Holland). The concentrations of calcium (Ca), magnesium (Mg), aluminum (Al), manganese (Mn), iron (Fe), zinc (Zn), copper (Cu), boron (B), nickel (Ni), molybdenum (Mo) and cobalt (Co) in the digestion solution were detected by inductively coupled plasma-mass spectrometry (ICP-MS 7900, Agilent Technologies, Santa Clara, California, US).

### Untargeted tea leaf metabolomics

Untargeted metabolomics methods were applied to tea leaves according to methods previously described by Chen et al. (2018) with minor modifications. Briefly, 50 mg ( ± 0.5 mg) of frozen tea sample powder was weighed into a 2 mL centrifuge tube prior to extracting metabolites in 1 mL of 70% (v/v) methanol solution (chromatographically pure). After vortexing, samples were sonicated at 25°C for 20 min and centrifuged at 12,000 g for 10 min prior to filtering supernatants through 0.22 µm PVDF filters and diluting filtrates 200-fold in 70% (v/v) methanol. Untargeted metabolomic analysis was then performed using ultra-high performance liquid chromatography-quadrupole-time-of-flight mass spectrometry (UPLC-Q-TOF/MS).

Data signal acquisition and analysis for the UPLC-Q-TOF/MS analysis system were performed with a Waters Acquity UPLC system coupled in tandem to a Waters photodiode array (PDA) detector and a SYNAPT G2-Si HDMS QTOF mass spectrometer (Waters, Manchester, UK). Chromatography was conducted by injecting 1 µL of extract into a Waters Acquity UPLC HSS T3 column (2.1 × 100 mm, 1.8 µm) running at 40°C and a flow rate of 0.3 mL/min. The mobile phases were water containing 0.1% formic acid (A) and acetonitrile containing 0.1% formic acid (B). The elution gradient was set to 99-93% A for the first two minutes, followed by 93-60% A for minutes 2-13, 60-1% A for minutes 13-14, and 1% A for minutes 14-17.

During mass spectrometry, electrospray ionization (ESI) was in the negative ion scanning mode, and the capillary voltage, cone voltage and collision energy were set to 2.5 kV, 40 eV and 4 eV, respectively. Additional settings at LC-MS runtime were as follows: source temperature was 120°C, desolvation temperature was 500°C, cone gas flow ran at 50 L/h, desolvation gas flow was set to 800 L/h, m/z range observed bracketed 50-1200 Da, and collision energy ranged from 10 to 50 eV. Leucine encephalin reference ions with m/z ratios of 554.2615 (for ESI^−^) were infused during data acquisition for calibration. Each of three replicated tea samples were analyzed once.

### Data processing and statistical analysis

Chromatograms from UPLC-QTOF-MS were processed using Progenesis QI software (version 2.1, Newcastle upon Tyne, UK) with default settings for peak picking, normalization, signal integration and initial peak assignments. A total of 717 metabolite signals were obtained and numbered FM1-717 for subsequent analysis, which used peak area abundance to represent the relative content of each metabolite. Metabolites were identified by comparing accurate masses, MS/MS fragmentation patterns and isotope patterns with authentic standards, as well as in consultation with the HMDB and Metlin online metabolite databases, and literature references (Tautenhahn et al., 2012; Chen et al., 2018; Wishart et al., 2022).

All data were expressed as means ± standard error (SE). Statistical significance of differences between pruned and unpruned samples were assessed using the Student’s *t*-test and two-way analysis of variance in SPSS software (Version 19.0, International Business Machines Corporation, Chicago, US). Metabolites significantly impacted by pruning were identified as those with variable importance in projection (VIP) > 1 and *P* value < 0.05. Graphs in this study were produced in Graphpad Prism software (Version 8.0, GraphPad Software, San Diego, US) and R (Version 3.6.2, Comprehensive R Archive Network, Shanghai, China). The randomForest function in R was used for random forest analysis (RF). The corrplot and igraph functions in R were used for correlation network analysis. The vegan and rdacca.hp functions in R were used for clustering analysis and redundancy analysis (RDA). Significantly impacted metabolites were annotated using the KEGG Compound database, mapped to KEGG pathways, and fed into metabolite set enrichment analysis. Heat maps were constructed using normalized data in the TBtools software (Version 1.068, https://github.com/CJ-Chen/TBtools (accessed on 15 April 2022), Guangzhou, China) (Chen et al., 2020).

## Results

### Pruning alters mineral nutrient components of tea leaves

**Figure 1:**
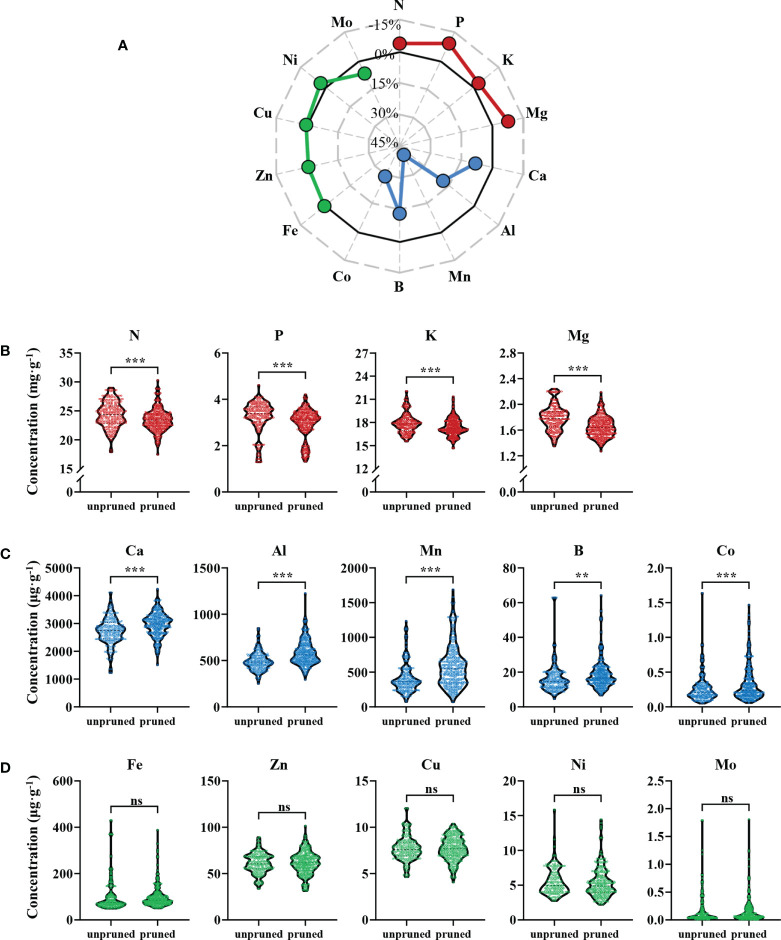
Ionomic analysis of fresh tea leaves from pruned and unpruned tea plants. **(A)** Percentage changes in mineral element concentrations with pruning. Percentage change = [(concentration_pruned_ - concentration_unpruned_)/concentration_unpruned_]×100%. Elements with concentrations that decreased **(B)**, increased **(C)** and did not change significantly **(D)** in fresh tea leaves from pruned tea plants. Values in the figure are the mean of 7 replicate samples per tea plantation (unpruned: n=132, pruned: n=218). Statistically significant differences between groups were tested by the Student’s *t* test. ***: *P* ≤ 0.001, **: 0.001 < *P* ≤ 0.01, ns, no significant difference.

Ionomic analysis of 2450 fresh tea leaf samples from 350 tea plantations revealed that pruning significantly affected mineral nutrient constituents of fresh tea leaves (**Figure 1**). The 14 observed mineral nutrients were divisible into three types according to their responses to pruning, which were those that were significantly reduced in concentrations (red dots), those were significantly increased in concentrations (blue dots), and those that were not significantly altered (green dots) after pruning (**Figure 1A**). Elements that declined with pruning were mainly N, P, K, and Mg, which were macro and medium elements. The concentrations of these four elements decreased by 3.65%, 9.03%, 2.81% and 7.76%, respectively. Among them, P had the largest decrease, followed by Mg (**Figure 1B**). Elements that were enhanced with pruning were all medium and micro elements, and included Ca, Al, Mn, B and Co, which increased by 8.16%, 18.66%, 40.45%, 13.02% and 29.08%, respectively. The concentration of Mn increased the most in pruned tea leaves (**Figure 1C**). Finally, the concentrations of five elements (Fe, Zn, Cu, Ni and Mo) were not significantly altered by pruning (**Figure 1D**).

These results show that pruning can significantly affect the distribution of multiple mineral nutrients in tea leaves. The concentrations of macro and medium elements, including N, P, K and Mg, decreased after pruning, while the concentrations of other medium and micro elements, including Ca, Al, Mn, B and Co, increased after pruning.

### Pruning tea plants affects activity of leaf secondary metabolic pathways

**Figure 2:**
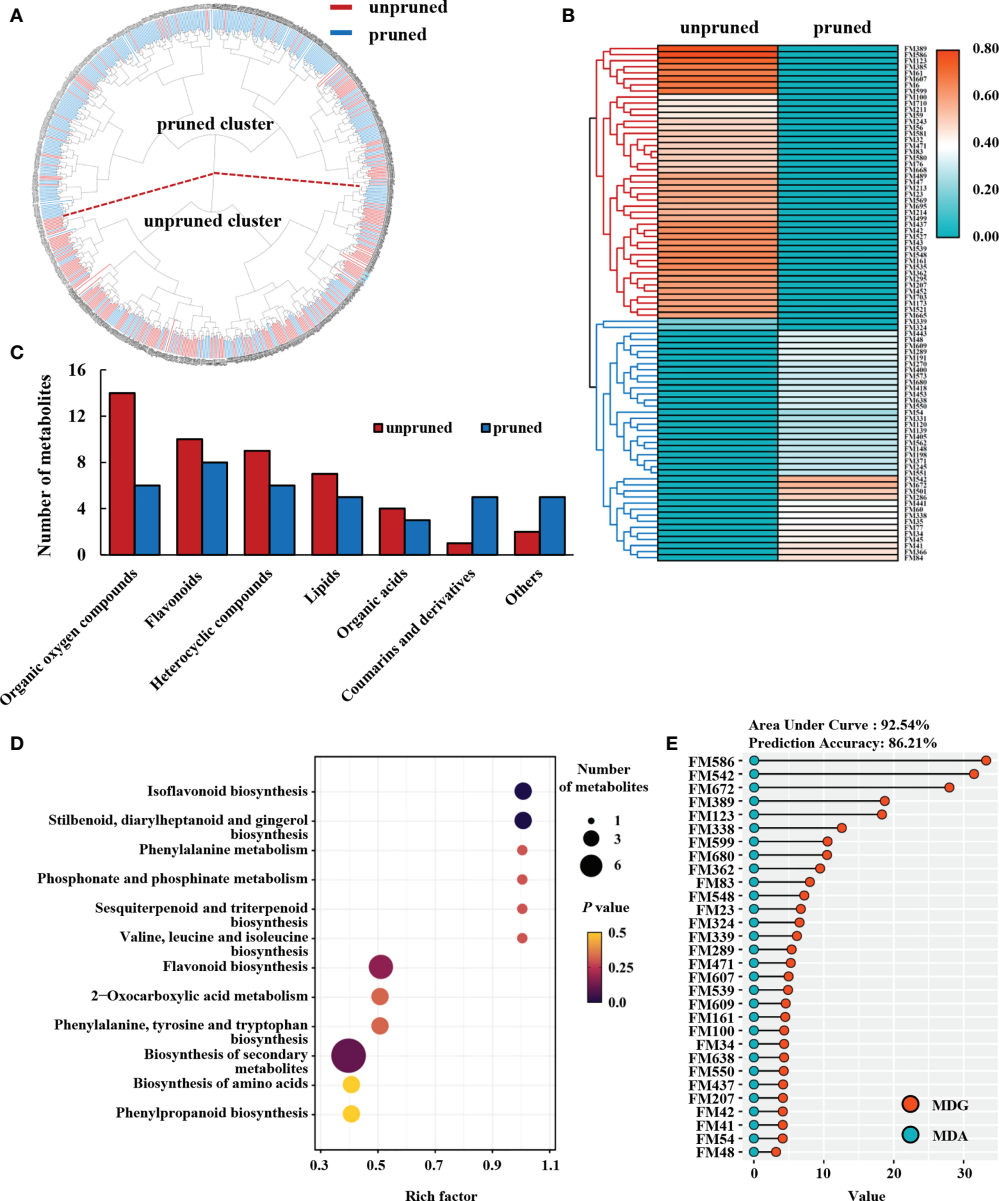
Metabolomic analysis of fresh tea leaves from pruned plants. **(A)** Cluster analysis of tea leaf samples. **(B)** Heatmap analysis of differential metabolites. Heat map values are normalized for the relative content of differential metabolites, and the average is calculated for each of the two pruning types of tea plant. **(C)** Class analysis of differential metabolites. **(D)** KEGG enrichment analyses of differential metabolites. **(E)** Random forest analysis of characteristic differential metabolites in fresh tea leaves. MDG, mean decrease Gini, MDA, mean decrease accuracy.

Cluster analysis was performed for 717 metabolite signals detected in the metabolome of tea samples under pruned and unpruned conditions. Considering each metabolite produced by the treatment, tea samples divided into two clusters, namely the pruned cluster and the unpruned cluster (**Figure 2A**). The proportion of pruned samples in the pruned cluster was 79.27%, and the proportion of unpruned samples in the unpruned cluster was 60.61% (**Figure 2A**).

The Student’s *t* test and partial least squares discriminant analysis (PLS-DA) were used to calculate the respective *P* values and variable importance in the projection (VIP) values of metabolites under pruned and unpruned conditions. According to the criteria of *P*<0.05 and VIP>1.0, 85 differential metabolites with database matches were finally obtained. Heat maps showed that differential metabolites divided into two distinct major branches (**Figure 2B**; **Supplementary Table 1**). One branch from unpruned plants contained 45, or 52.94% of the total differential metabolites, while the other branch from pruned tea plants contained 38 (44.71% of the total) differential metabolites (**Figure 2B**).

Closer inspection revealed that differential metabolites were largely one of seven chemical categories, either organic oxygen compounds, flavonoids, heterocyclic compounds, lipids, organic acids, coumarins and derivatives, and other compounds. The number of coumarins and derivatives accumulating in leaves from pruned tea plants was significantly higher than in leaves from unpruned plants, while significantly higher numbers of organic oxygen compounds, flavonoids, heterocyclic compounds, lipids and organic acids accumulate in unpruned tea leaves than in pruned tea leaves. Differences in accumulation were most pronounced for organic oxygen compounds, with 57.14% less in leaves from pruned tea plants than in leaves of unpruned tea plants (**Figure 2C**).

KEGG enrichment analysis showed that differential metabolites enhanced by pruning fell mainly into 12 enriched metabolic pathways, including isoflavonoid biosynthesis, stilbenoid, diarylheptanoid and gingerol biosynthesis, phenylalanine metabolism, phosphonate and phosphinate metabolism, sesquiterpenoid and triterpenoid biosynthesis, valine, leucine and isoleucine biosynthesis, and flavonoid biosynthesis (**Figure 2D**). This suggests that pruning affects secondary metabolic pathways of subsequently produced tea leaves, and thereby changes the contents of flavonoids, amino acids and terpenoids.

The 85 identified differential metabolites were analyzed by random forest methods, and the top 30 metabolites with the largest differential contributions were selected based on a combination of accuracy and mean decrease in Gini impact (**Figure 2E**). These 30 metabolites were thusly regarded as the important metabolites that were characteristic of production in tea leaves after pruning. The area under curve (AUC) and the prediction accuracy (PA) of the predictive model for pruned and unpruned samples based on the top 30 differential metabolites reached 92.54% and 86.21%, respectively (**Figure 2E**). The top 30 characteristic metabolites selected through random forest analysis were, therefore, indicative of changes in tea secondary metabolism observed after pruning to a large extent.

The above results indicate that pruning may significantly impact the enrichment of metabolites in tea leaves, with the most prominent effects noted here for organic oxygen compounds and flavonoids which suggests that pruning promotes specific secondary metabolic pathways in subsequently produced tea leaves.

### Key mineral nutrients and secondary metabolites affected by pruning

**Figure 3:**
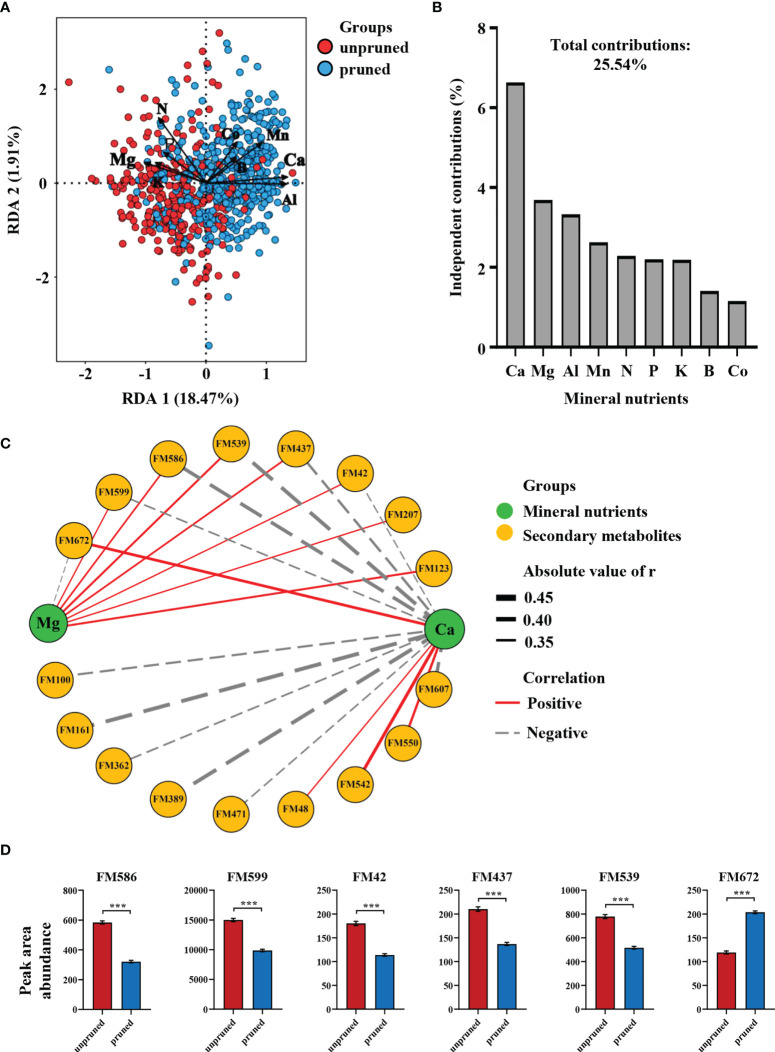
**(A)** Redundancy analysis of mineral elements and characteristic differential metabolites under pruned conditions. **(B)** Independent contributions of mineral elements on metabolite differences. **(C)** Correlation network analysis of calcium and magnesium with characteristic metabolites. **(D)** Key metabolites regulated by calcium and magnesium. Statistically significant differences between groups were tested by the Student’s *t* test. ***P ≤ 0.001.

To further explore connections between tea plant pruning effects on mineral nutrients and changes in secondary metabolism, 30 characteristic differential metabolites and nine differential mineral elements were subjected to redundancy analysis (RDA). Here, pruned samples were mostly distributed in the positive semi-axis direction of RDA1, while unpruned samples were mostly concentrated in the negative semi-axis direction of RDA1. RDA1 and RDA2 together accounted for 20.38% of the variation among tea samples (**Figure 3A**). The distribution of pruned samples was positively correlated with Ca, Al, and Mn, while unpruned samples correlated with Mg and K (**Figure 3A**), which was consistent with variations in nutrient element concentrations displayed in **Figure 1B, C**. The total contribution of the nine mineral elements to the differences in characteristic metabolites produced after pruning was 25.54% (**Figure 3B**). The contributions of the top four elements ranked in order were 6.64% for Ca, 3.69% for Mg, 3.33% for Al and 2.63% for Mn. Notably, the two divalent cations Ca and Mg affected the characteristic metabolites in opposite directions, and the sum of their independent contributions accounted for more than 40% of the total differential contributions (**Figures 3A, B**). This result suggests that changes in mineral nutrient concentrations are important determinants of alterations in secondary metabolites produced after pruning, with Ca and Mg elements appearing to play a central role.

Network correlation analysis of Ca and Mg with 30 characteristic metabolites revealed that a total 17 secondary metabolites exhibited significant correlations with both elements (**Figure 3C**). Fifteen secondary metabolites were significantly correlated with Ca, of which 11 exhibited negative correlations, while 8 secondary metabolites were significantly correlated with Mg, seven of which were positive correlations (**Figure 3C**). These results implicate Ca as the key element in determining the set of characteristic differential metabolites produced after pruning, with Mg also playing an important role primarily in opposition to Ca.

Further focus on characteristic metabolites co-regulated by Ca and Mg revealed five that were negatively regulated by Ca, including 1,3-dicaffeoylquinic acid (FM586), Neryl arabinofuranosyl-glucoside (FM599), Hyaluronic acid (FM42), Valyl-asparagine (FM437) and a β-hydroxy ketone compound (FM539). Only 2-O-caffeoyl arbutin (FM672) was positively regulated by Ca (**Figure 3C**). It is also noteworthy that the top three characteristic metabolites identified in random forests included 2-O-caffeyl arbutin and 1,3-dicaffeoylquinic acid, which increased by 71.07% and decreased by 45.02%, respectively in tea leaves after pruning (**Figure 3D**).

The results outlined above indicate that changes in tea leaf mineral nutrients after pruning are closely related to differences in secondary metabolites. The most important elements affecting metabolite concentrations in tea leaves were Ca and Mg, which had opposite effects on six co-regulated characteristic metabolites, including 1,3-dicaffeoylquinic acid and 2-O-caffeyl arbutin.

### Effects of pruning on key mineral nutrients and secondary metabolites of tea samples from representative sites

To further verify whether key mineral nutrients and characteristic metabolites screened from multiple tea plantations were affected by soil fertility status and growth environment, this study included a two-way ANOVA of soil fertility properties on a total of 24 tea plantations in six representative sites (**Table 1**). Results indicated that there was no interaction between the seven soil fertility properties under the two factors (site and pruning), with only site producing significant differences between pruning treatments (**Table 1**). This indicated that soil fertility status varies widely across the six independent representative sites. In general, the soils were acidic, with pH values of 4.31-5.13 observed. The ranges of soil OM, AN, AP, AK, E-Ca and E-Mg were 17.88-39.1 g·kg^-1^, 69.07-142.22 mg·kg^-1^, 6.41-312.71 mg·kg^-1^, 77.96-239.28 mg·kg^-1^, 111.92-925.67 mg·kg^-1^ and 27.37-136.65 mg·kg^-1^, respectively (**Table 2**). There were no significant differences in soil fertility properties between pruned and unpruned tea plantations within sites (**Table 2**), which indicated that soil fertility and cultivation management were relatively consistent between pruned and unpruned tea plantations at representative sites.

**Table 1:** Tow-way ANOVA of soil fertility properties in representative sites.

	Factors		
Soil properties	Site	Pruning	Site X Pruning
pH	7.207 ***	0.192 ns	0.888 ns
OM	8.966 ***	0.535 ns	0.738 ns
AN	9.210 ***	2.227 ns	1.323 ns
AP	9.634 ***	1.800 ns	1.479 ns
AK	13.457 ***	1.318 ns	0.447 ns
E-Ca	6.888 ***	0.312 ns	1.662 ns
E-Mg	13.061 ***	0.089 ns	1.960 ns

Data in the table are *F*-values of two-way ANOVA. ***P ≤ 0.001, ns, no significant difference.

**Table 2:** Soil fertility properties of tea plantations in representative sites.

	Soil properties							
Type of tea	plantations	pH	OM (g·kg^-1^)	AN (mg·kg^-1^)	AP (mg·kg^-1^)	AK (mg·kg^-1^)	E-Ca (mg·kg^-1^)	E-Mg (mg·kg^-1^)
	unpruned	4.53+0.04	23.12+1.42	94.91+4.86	219.67+71.84	107.4+8.23	322.52+25.85	60.4+4.69
Sitel	pruned	4.76:0.16	23.4+3.08	80.27+6	171.28+28.85	106.51+5.06	438.27+53.54	91.43+13.44
	*P* value	0.192 ns	0.937 ns	0.087 ns	0.546 ns	0.928 ns	0.080 ns	0.054 ns
	unpruned	4.95+0.34	25.39+1.76	97.07+7.59	116.44+29.59	239.28+14.97	925.67+328.21	136.65+24.75
Site2	pruned	4.69+0.1	20.95+1.28	80.73±7.61	253.7+62.03	199.67+10.66	466.86+75.52	86.28+17.41
	*P* value	0.439 ns	0.068 n	0.160 ns	0.063: ns	0.062 ns	0.203 ns	0.127 ns
	unpruned	4.7+0.21	22.77+4.16	75.02+12.12	74.34+5.06	130.05+11.96	420.4+91.37	45.64+5.33
Site3	pruned	4.69+0.07	25.94+1.14	69.07=2.95	91.03:27.44	111.5+10.85	457.58+55.41	44+5.91
	*P* value	0.976 ns	0.480 ns	0.644 ns	0.563 ns	0.278 n	0.735 ns	0.841 ns
	unpruned	4.44+0.04	22.69+1.99	76.42=7.8	173.65+59.95	146.09+12.19	128.08=20.31	37.63+7.25
Site4	pruned	4.31+0.05	29.27+2.94	84.93+3.85	312.71+78.75	128.6+7.25	182.14+22.35	33.84+2.85
	*P* value	0.075 ns	0.093 ns	0.350 ns	0.190 ns	0.246 ns	0.104 ns	0.637 n
	unpruned	4.61+0.09	38.13+1.54	142.22+12.43	6.41+1.05	77.96+16.3	174.66+43.25	27.37+4.29
Site5	pruned	4.55+0.07	39.1+7.39	118.42+18.32	17.99+5.45	81.63+19.89	111.92+14.75	33.66:8.04
	*P* value	0.633 ns	0.900 ns	0.308 ns	0.051 n	0.890 ns	0.200 ns	0.506 ns
	unpruned	5.13+0.04	17.88+2.97	89.37+7.04	7.57=2.24	148.38=29.75	261.07+30.54	53.05+4.47
Site6	pruned	5.12+0.04	21.25+2.65	80.62+10.66	10.15+1.64	108.76+26.84	318.17+86.62	54.66+12.87
	*P* value	0.890 ns	0.416 ns	0.509 ns	0.375 ns	0.346 ns	0.548 ns	0.908 ns

Values are shown as means ± standard errors. Testing for statistically significant differences between groups were conducted using the Student’s *t* test. ns, no significant difference.

**Figure 4:**
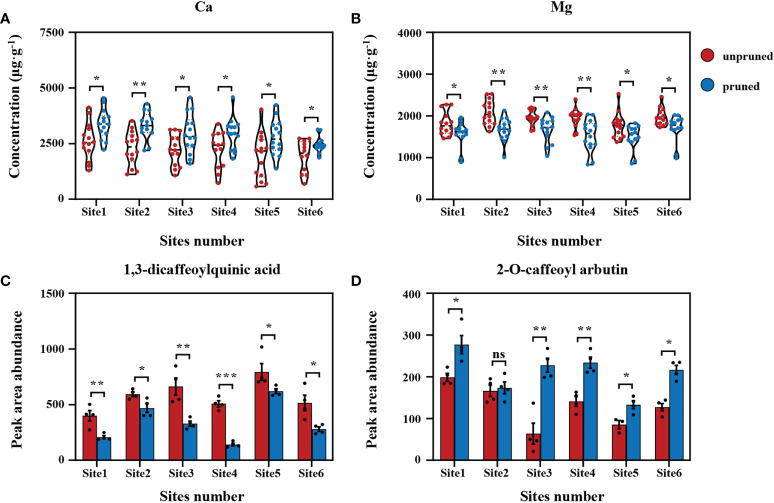
Difference analysis of fresh tea leaf samples from representative sites. Comparisons of calcium **(A)** and magnesium **(B)** concentrations in fresh tea leaves from representative sites. Comparisons of 1,3-dicaffeoylquinic acid **(C)** and 2-O-caffeoyl arbutin **(D)** relative contents in fresh tea leaves from representative sites. Values are shown as means ± standard errors. Statistically significant differences between groups were tested by the Student’s *t* test. ***: *P* ≤ 0.001, **: 0.001 < *P* ≤ 0.01, *: 0.01 < *P* ≤ 0.05, ns: no significant difference.

To sharpen focus on critical determinants of tea quality, differential analysis was performed on data collected from the six representative locations for the key tea mineral nutrients Ca and Mg and the selected significant secondary metabolites 1,3-dicaffeoylquinic acid and 2-O-caffeoyl arbutin. Here, it was found that the concentration of Ca and the relative content of 2-O-caffeoyl arbutin in tea leaves increased significantly after pruning, while the concentration of Mg and the relative content of 1,3-dicaffeoylquinic acid decreased significantly (**Figure 4**), which was consistent with data collected from the entirety of tea plantations (**Figures 1B, C**; **Figure 3D**). In short, the changes observed for key mineral nutrients and secondary metabolites in response to pruning across otherwise consistent plantations were consistent with the results of surveys over all observed tea plantations. This clearly indicates that pruning tea plants plays a dominant role in influencing metabolism and quality components, although soil fertility varies greatly.

## Discussion

### Pruning affects the accumulation and proportions of tea leaf mineral nutrients

Pruning is an important tea plants management strategy that can increase yields when correctly applied (Mohale et al., 2018; Thakur et al., 2019; Botelho et al., 2020). Conversely, long-term unpruned growth can significantly change the flavor characteristics of tea products (Chen et al., 2021). The mineral nutrients required for the growth and development of tea plants are mainly obtained from the soil through the root system, and then transported to the shoot through the xylem (Barberon and Geldner, 2014). N, P, K and Mg are highly mobile in the phloem, and can also be transported from source to sink, from old leaves to young leaves, for the growth and development of young leaves (White, 2012; Pongrac et al., 2020). Therefore, pruning may affect the transport and distribution of mineral nutrients in tea plants. Leaf concentrations of four elements, N, P, K and Mg, decreased significantly with pruning of tea plants in this study (**Figure 1B**). The reason may be that pruning prevents the redistribution of these elements from old source leaves to young sink leaves through phloem connections, and thereby reduces a source of nutrients for subsequently harvested tea leaves (White, 2012; Peng et al., 2019).

Pruning may disrupt apical dominance, promote adventitious bud germination, and increase later yields (Balandier et al., 2000; Qin et al., 2019). One tradeoff is that the large amount of mineral nutrients contained in pruned branches is lost to remobilization, making nutrient depletion more likely (Kaba and Abunyewa, 2021), especially for the highly demanded mobile elements N, P, K and Mg. Tea plants also store considerable amounts of mineral nutrients in roots that are a primary source of nutrients for annual vegetative growth. Pruning may deplete root system reserves, and depress mineral absorption and translocation to a certain extent (Kang et al., 2019). Declines in root nutrient storage levels, therefore, may lead to reductions in leaf N, P, K and Mg concentrations.

Pruning disrupts the balance of resource allocation and distribution of growth among plant tissues, which alters the capacity to assimilate and distribute nutrients (Kang et al., 2019). Poplar, for one, may respond to pruning with decreases in leaf N, P and K contents (Jing et al., 2018), which is consistent with our results. Plus, tea is known to respond to pruning by altering translocation of these mineral nutrients to accumulate more in roots at the expense of shoots (Arkorful et al., 2020a). With N, Mg and P acting as basic constituents of chlorophyll and energy metabolism, the accumulation of these elements in higher concentrations in unpruned tea leaves in comparison to leaves on pruned plants may lead to differences in photosynthesis. Chen et al. (2021) made observations consistent with this hypothesis when they found that leaves of unpruned tea plants exhibited long term significant increases in chlorophyll a and b contents, along with enhanced photosynthesis relative to leaves on pruned cohorts.

Our investigation also revealed that, in contrast to the decline of specific mobile elements, Ca, Al, Mn, B, and Co instead accumulated significantly in leaves of pruned tea plants relative to leaves on unpruned plants (**Figure 1C**). On one hand, these elements may accumulate with pruning because pruning shortens the transport distance of nutrients in the xylem, so mineral nutrients can accumulate in tea leaves for some time as driven by the upward force of transpiration (White, 2012). This implies that leaves with stronger transpiration accumulate more of these five nutrients. Alternatively, accumulation with pruning might be related to phloem mobility, especially the low phloem mobilities of Ca and Mn (Pongrac et al., 2020). This makes them difficult to redistribute from old leaves to young leaves, and with more leaves on unpruned plants than pruned plants, the amount that does reach each unpruned tea leaf is relatively low.

It is worth noting that Sun et al. (2020) found that Al is an essential element for the growth and development of tea root systems, with most of the absorbed Al accumulating in roots and old leaves. However, less information is available on the mobility of Al in tea plants. In addition, the mobility of Al, and other nutrients for that matter, might vary among plant species. In this study, the mobility of Al, B and Co appeared to be very low in both pruned and unpruned tea plants.

### Pruning alters the enrichment of secondary metabolites in tea leaves

Tea taste and aroma are determined by secondary metabolites (Ho et al., 2015; Das et al., 2019) that vary across seasons, varieties, regions, fertilization practices, processing technologies and many other factors (Chen et al., 2018; Dong et al., 2019; Rubel Mozumder et al., 2021; Liu et al., 2022). Pruning is widely reported as a method for improving the quality of crops and fruits, such as grapes and roses (Gatti et al., 2011; Thakur et al., 2019). Rubel Mozumder et al. (2021) reported that glucose, catechins and their derivatives were significantly higher in unpruned compared to pruned tea leaves, which may be related to the availability of nutrients to remobilize and support photosynthesis in leaves of unpruned tea plants as described above. In addition, Sun et al. (2018) identified impacts of long-term tea plant pruning on catechins through metabolomic and transcriptomic techniques, with epigallocatechin gallate (EGCG) and caffeine notably lower in leaves of unpruned tea plants relative to leaves of pruned plants, which leads to less astringency and bitterness of the commercial tea produced from unpruned plants. Chen et al. (2021) used the sensory evaluation panels to find that tea leaves from unpruned plants had the best aroma and taste, and biochemical assays yielded complementary results in which the amino acid and aromatic compound contents were significantly higher in leaves of unpruned tea plants than in leaves of pruned tea plants. These differences in amino acids, aromatic compounds and secondary metabolites may be a primary reason why tea from unpruned tea plants is generally considered the most fresh and mellow.

Experiments herein revealed that pruning may alter metabolic profiles in fresh tea leaves (**Figure 2A, B**), with fresh leaves of unpruned plants accumulating the most metabolites, predominantly organic oxygen compounds, flavonoids and heterocyclic compounds (**Figure 2C**). Phenolic glycosides mainly accumulated in leaves of pruned tea plants, while quinic acid and its derivatives, as well as, 9 flavonoid glycosides mainly accumulated in leaves of unpruned tea plants. This is consistent with Arkorful et al. (2020b) in that flavonoid glycosides appeared to change dramatically upon pruning.

Environmental stress has been reported to stimulate accumulation of phenylpropanoids in plants (Dixon and Paiva, 1995). Plus, increased oxidative stress in pruned tea plants often leads to increases of antioxidant compounds, such as phenolic compounds, which originate from the phenylpropanoid metabolic pathway (Mahanta and Baruah, 1992; Goufo et al., 2014). These pruning induced secondary metabolites might help tea plants adapt to environmental challenges and overcome stress. Quinic acid is one of the precursors in the production of phenylalanine, tyrosine and tryptophan (Carrington et al., 2018), which further indicates that amino acid metabolism is more pronounced in unpruned than in pruned tea plants. In our observations, the differential metabolites enriched by pruning were mainly flavonoids, phenylpropanoids and amino acids (**Figure 2D**), which suggests upregulation of these metabolic pathways and is consistent with previous reports.

### Mineral nutrients affect tea quality characteristics

Chemical composition is a key factor in determining the quality of tea (Rubel Mozumder et al., 2021). Among chemical constituents, caffeine, catechin, and its derivatives determine the bitterness and astringency of tea infusions (Han et al., 2016), while amino acids endow tea infusions with freshness (Das et al., 2019). Organic acids and soluble sugars add acidity and sweetness to tea infusions (Chen et al., 2011).

Mineral nutrients are required to synthesize and regulate the biochemical components of tea quality. Sun et al. (2019) found that the concentrations of N, P and K in fresh tea leaves were positively correlated with theanine and caffeine production, and negatively correlated with the concentration of non-galloylated catechins. Focusing on direct metabolic connections, Huang et al. (2018) found that under different N source conditions, the accumulation of flavonoids and amino acids varied through regulation of the distribution of N through tea plants. In short, existing literature agrees that mineral nutrition is a primary determinant of tea flavor.

In our more comprehensive approach, pruning significantly altered the distribution and accumulation of numerous mineral nutrients in tea leaves, though Ca and Mg were most closely, and oppositely associated with the metabolites produced upon pruning (**Figure 3B, C**). Pruning favored the accumulation of Ca, and reduced the accumulation of Mg (**Figure 1A, B**), and the effects on characteristic metabolite accumulation followed these opposing trends. Among the six key characteristic metabolites co-regulated by Ca and Mg, 2-O-caffeyl arbutin was positively regulated by Ca, while 1,3-dicaffeoylquinic acid was positively regulated by Mg (**Figure 3D**). Caffeoyl arbutin is a glycoside formed from a class of phenolic compounds that are enriched in Quezui tea, with its astringent and bitter tastes (Zhao et al., 2008). As an alicyclic organic acid, 1,3-dicaffeoylquinic acid is an important precursor for the synthesis of aromatic amino acids in teas with a certain weak acidity (Danino et al., 2009; Carrington et al., 2018). The high accumulation of quinic acid and its derivatives in leaves of unpruned tea plants may be beneficial for promoting the synthesis of aromatic amino acids that endow tea with freshness qualities (Chen et al., 2021).

Through long diversification processes, tea plants have formed their own ecological adaptation types (Liang et al., 2021). For one, tea adapted to grow in calcium-deficient acidic soil by demanding relatively low amounts of Ca, and thusly is considered a calcifuge plant (Liang et al., 2021). Pruning shortens the transport distance of nutrients, allowing Ca to accumulate in tea leaves where it may promote the accumulation of phenolic glycosides, which is consistent with previous reports that Ca can regulate sugar metabolism in sugar beet (Hosseini et al., 2019). It has also been widely reported that Ca ion signaling plays important roles in plant stress responses (Ding et al., 2019), and that phenolic compounds play important roles in plant protection, mainly in preventing damage caused by biotic and abiotic stressors (Goufo et al., 2014; Dong et al., 2019). The increase of Ca and phenolic glycosides in tea leaves after pruning may, therefore, be a crucial mechanism for coping with pruning stress. Unpruned tea plants, in contrast, have wider canopies and need to produce more photosynthetic products for the trees to grow. Therefore, accumulation of Mg in unpruned tea leaves is beneficial for chlorophyll synthesis, increasing Rubisco activity, and enhancing net photosynthesis (Peng et al., 2019). This, in turn, promotes the photorespiration cycle and the shikimic acid pathway, which leads to the accumulation of quinic acid and its derivatives (Shao et al., 2021) that enrich tea with sour and refreshing tastes. Overall, Ca and Mg are the most abundant divalent cations in plants, and they often have antagonistic effects in plant cells, as we discovered with pruning of tea plants. Therefore, regulating the homeostatic balance between Ca and Mg is vital for promoting the most conducive conditions for tea plant growth and development (Tang and Luan, 2017). Pruning of tea plants in our experiments changed the distribution and accumulation of Ca and Mg concentrations in subsequently harvested leaves, and, thereby, may endow tea infusions with unique quality characteristics for leaves from pruned and unpruned tea plants.

Environmental factors, plantation management, and regulation of tea plant growth all have dramatic influences on tea quality (Ding et al., 2019; Dong et al., 2019). In particular, soil, as the main source of mineral nutrients, directly determines the abundance and deficiency of mineral nutrients in tea leaves. Comparing pruned and unpruned tea samples from six representative sites exhibiting large differences in soil fertility (**Table 1**) revealed that changes in tea leaf Ca and Mg concentrations, along with associated effects on 1,3-dicaffeoylquinic acid and 2-O-caffeoyl arbutin relative contents were all consistent across multiple tea plantations (**Figure 4**). This indicates strong heritability in pruning responses, with pruning playing a leading role in influencing key mineral nutrients and their characteristic metabolites, even across large differences in soil fertility.

## Conclusion

Through ionomic and metabolomic analysis of 1962 fresh tea leaf samples from pruned plants and 1188 from unpruned plants, observations of the main cultivar reared to produce Wuyi rock tea, Shuixian, revealed that pruning can significantly affect the distribution and accumulation of mineral nutrients in tea leaves. Pruning favored the accumulation of less mobile mineral nutrients, while withholding pruning enhanced leaf contents of mobile nutrients. At the same time, pruning may promote enrichment secondary metabolites in tea leaves. Overall, Ca and Mg are important elements that appear to act in opposition in effects on tea characteristic metabolites (such as 1,3-dicaffeoylquinic acid and 2-O-caffeoyl arbutin). In summary, pruning may affect the distribution and transportation of mineral nutrients in tea plants, and the concomitant accumulation of key characteristic metabolites, and thereby affecting the quality of tea leaves. The results of this study provide a theoretical basis for designing suitable tea plantation cultivation and management practices to enhance desired qualities of harvested tea.

## Data availability statement

The original contributions presented in the study are included in the article/**Supplementary Material**. Further inquiries can be directed to the corresponding author.

## Author contributions

HL designed the experiments and critically revised the manuscript. YL analyzed the data and wrote the original manuscript. YL, JT, BeL, ZZ, CS, RX, LS carried out the investigations and experiments. MX, BaL, JY provided resources. All authors have read and agreed to the published version of the manuscript. All authors contributed to the article and approved the submitted version.

## Funding

This research was financially supported by MOA Modern Agricultural Talents Support Project.

## Acknowledgments

We would like to acknowledge the students of the Root Biology Center, Fujian Agriculture and Forestry University for tea and soil sample collection, and Dr. Thomas Walk of Golden Fidelity LLC for his careful revision on this paper.

## Conflict of interest

The authors declare that the research was conducted in the absence of any commercial or financial relationships that could be construed as a potential conflict of interest.

## Publisher’s note

All claims expressed in this article are solely those of the authors and do not necessarily represent those of their affiliated organizations, or those of the publisher, the editors and the reviewers. Any product that may be evaluated in this article, or claim that may be made by its manufacturer, is not guaranteed or endorsed by the publisher.

## Supplementary material

Supplementary Figure 1 Map of tea plantation locations and representative sties.
