# Supplementary figures and images for: Effects of pruning on mineral nutrients and untargeted metabolites in fresh leaves of Camellia sinensis cv. Shuixian
Source: Front Plant Sci. 2022 Oct 13;13:1016511. doi: 10.3389/fpls.2022.1016511 (PMC9606708; doi:10.3389/fpls.2022.1016511)

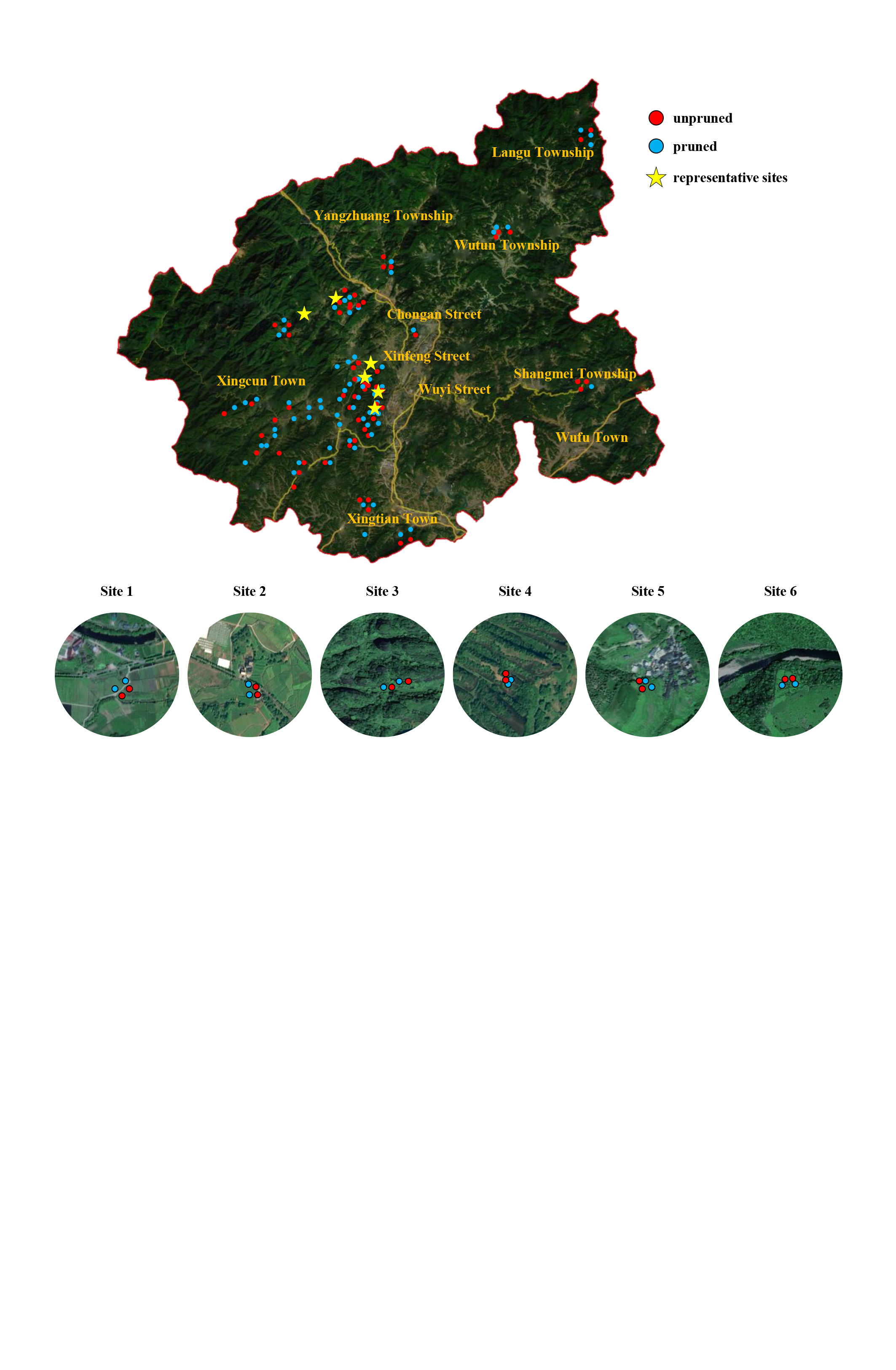

Supplement: Supplementary Figure 1 — Map of tea plantation locations and representative sties. [file Image_1.jpeg]
